# Summing $\mu (n)$: a faster elementary algorithm

**DOI:** 10.1007/s40993-022-00408-8

**Published:** 2022-12-08

**Authors:** Harald Andrés Helfgott, Lola Thompson

**Affiliations:** 1 Mathematisches Institut, Georg-August Universität Göttingen, Bunsenstraße 3-5, 37073 Göttingen, Germany; 2 IMJ-PRG, UMR 7586, 58 Avenue de France, Bâtiment S. Germain, case 7012, 75013 Paris Cedex 13, France; 3 Mathematics Institute, Utrecht University, Hans Freudenthalgebouw, Budapestlaan 6, 3584 CD Utrecht, Netherlands

## Abstract

We present a new elementary algorithm that takes $\textrm{time} \ \ O_\epsilon \left( x^{\frac{3}{5}} (\log x)^{\frac{8}{5}+\epsilon } \right) \ \ \textrm{and} \ \textrm{space} \ \ O\left( x^{\frac{3}{10}} (\log x)^{\frac{13}{10}} \right)$ (measured bitwise) for computing $M(x) = \sum _{n \le x} \mu (n),$ where $\mu (n)$ is the Möbius function. This is the first improvement in the exponent of *x* for an elementary algorithm since 1985. We also show that it is possible to reduce space consumption to $O(x^{1/5} (\log x)^{5/3})$ by the use of (Helfgott in: Math Comput 89:333–350, 2020), at the cost of letting time rise to the order of $x^{3/5} (\log x)^2 \log \log x$.

## Introduction

There are several well-studied sums in analytic number theory that involve the Möbius function. For example, Mertens [14] considered

$$\begin{aligned} M(x) = \sum _{n \le x} \mu (n), \end{aligned}$$

now called the *Mertens function*. Based on numerical evidence, he conjectured that $|M(x)| \le \sqrt{x}$ for all *x*. His conjecture was disproved by Odlyzko and te Riele [16]. Pintz [17] made their result effective, showing that there exists a value of $x < \exp (3.21 \times 10^{64})$ for which $|M(x)| > \sqrt{x}$. It is still not known when $|M(x)| > \sqrt{x}$ holds for the first time; Dress [2] has shown that it cannot hold for $x\le 10^{12}$, and Hurst has carried out a verification up to $10^{16}$ [6]. Isolated values of *M*(*x*) have been computed in [2] and in subsequent papers.

The two most time-efficient algorithms known for computing *M*(*x*) are the following:

1. An analytic algorithm (Lagarias-Odlyzko [13]), with computations based on integrals of $\zeta (s)$; its running time is $O(x^{1/2+\epsilon })$.
2. A more elementary algorithm (Meissel-Lehmer [10] and Lagarias-Miller-Odlyzko [12]; refined by Deléglise-Rivat [1]), with running time about $O(x^{2/3})$.

These algorithms are variants of similar algorithms for computing $\pi (x)$, the number of primes up to *x*. The analytic algorithm had to wait for almost 30 years to receive its first rigorous, unconditional implementation due to Platt [18], which concerns only the computation of $\pi (x)$. The computation of *M*(*x*) using the analytic algorithm presents additional complications and has not been implemented. Moreover, in the range explored to date ($x\le 10^{22}$), elementary algorithms are faster in practice, at least for computing $\pi (x)$.

Deléglise and Rivat’s paper [1] gives the values of *M*(*x*) for $x=10^6,10^7,\dotsc ,10^{16}$. An unpublished 2011 preprint of Kuznetsov [9] gives the values of *M*(*x*) for $x=10^{16},10^{17},\dotsc ,10^{22}$ using parallel computing. More recently, Hurst [6] computed *M*(*x*) for $x = 2^n$, $n\le 73$. (Note that $2^{73} = 9.444\dotsc \cdot 10^{21}$.) The computations in [9] and [6] are both based on the algorithm in [1].

Since 1996, all work on these problems has centered on improving the implementation, with no essential improvements to the algorithm or to its computational complexity. The goal of the present paper is to develop a new elementary algorithm that is more time-efficient and space-efficient than the algorithm in [1]. We show:

### MainTheorem

We can compute *M*(*x*) in

$$\begin{aligned} \begin{aligned}&O\left( x^{\frac{3}{5}} (\log x)^{\frac{3}{5}} (\log \log x)^{\frac{2}{5}} \right) \;\;\text {word operations,}\\ \text {time} \;\;&O\left( x^{\frac{3}{5}} (\log x)^{\frac{8}{5}} (\log \log x)^{\frac{7}{5}} \right) ,\\ \text {and space} \;\;&O\left( x^{\frac{3}{10}} (\log x)^{\frac{13}{10}} (\log \log x)^{-\frac{3}{10}} \right) .\end{aligned} \end{aligned}$$

Space here is measured in bits, and time is measured in bit operations. “Word operations” (henceforth “operations”) means arithmetic operations ($+$, −, $\cdot$, /, $\surd$) on integers of absolute value up to $x^{O(1)}$, as well as memory operations (read and write) in arrays of such integers with indices up to $x^{O(1)}$. Some of the literature (including both [1] and earlier versions of the present paper) counts time in terms of word operations; some (e.g., [13]) makes it clear that it counts bit operations.

Ours is the first improvement in the exponent of *x* since 1985. Using our algorithm, we have been able to extend the work of Hurst and Kuznetsov, computing *M*(*x*) for $x = 2^n$, $n\le 75$, and for $x = 10^{n}$, $n\le 23$. We expect that professional programmers who have access to significant computer resources will be able to extend this range further.

### Our approach

The general idea used in all of the elementary algorithms ([1, 12], etc.) is as follows. One always starts with a combinatorial identity to break *M*(*x*) into smaller sums. For example, a variant of Vaughan’s identity allows one to rewrite *M*(*x*) as follows:

$$\begin{aligned} M(x) = 2M(\sqrt{x}) - \sum _{n \le x} \sum _{\begin{array}{c} m_1 m_2 n_1 = n \\ m_1, m_2 \le \sqrt{x} \end{array}} \mu (m_1) \mu (m_2). \end{aligned}$$

Swapping the order of summation, one can write

$$\begin{aligned} M(x) = 2M(\sqrt{x}) - \sum _{m_1, m_2 \le \sqrt{x}} \mu (m_1) \mu (m_2) \left\lfloor \frac{x}{m_1 m_2} \right\rfloor . \end{aligned}$$

The first term can be easily computed in $O(\sqrt{x} \log \log x)$ operations and space $O(x^{1/4})$, or else, proceeding as in [5], in $O(\sqrt{x} \log x)$ operations and space $O(x^{1/6} (\log x)^{2/3})$. To handle the subtracted term, the idea is to fix a parameter $v \le \sqrt{x}$, and then split the sum into two sums: one over $m_1, m_2 \le v$ and the other with $\max (m_1, m_2) > v$. The difference between the approach taken in the present paper and those that came before it is that our predecessors take $v = x^{1/3}$ and then compute the sum for $m_1, m_2 \le v$ in $O(v^2)$ operations. We will take our *v* to be a little larger, namely, about $x^{2/5}$. Because we take a larger value of *v*, we have to treat the case with $m_1, m_2 \le v$ with greater care than [1] et al. Indeed, the bulk of our work will be in Sect. 4, where we show how to handle this case.

Our approach in Sect. 4 roughly amounts to analyzing the difference between reality and a model that we obtain via Diophantine approximation, in that we show that this difference has a simple description in terms of congruence classes and segments. This description allows us to compute the difference quickly, in part by means of table lookups.

### Alternatives

In a previous draft of our paper, we followed a route more closely related to the main ideas in papers by Galway [3] and by the first author [5]. Those papers succeeded in reducing the space needed for implementing the sieve of Eratosthenes (or the Atkin-Bernstein sieve, in Galway’s case) down to about $O(x^{1/3})$. In particular, [5] provides an algorithm for computing $\mu (n)$ for all successive $n\le x$ in $O(x \log x)$ operations and space $O(x^{1/3} (\log x)^{2/3})$, building on an approach from a paper of Croot, Helfgott, and Tao [19] that computes $\sum _{n\le x} \tau (n)$ in about $O(x^{1/3})$ operations. That approach is in turn related to Vinogradov’s take on the divisor problem [20, Ch. III, exer. 3-6] (based on Voronoï).

The total number of word operations taken by the algorithm in the previous version of our paper was on the order of $x^{3/5} (\log x)^{8/5}$. Thus, the current version is asymptotically faster. If an unrelated improvement present in the current version (Algorithm 23; see Sect. 3) were introduced in the older version, the number of word operations would be on the order of $x^{3/5} (\log x)^{6/5} (\log \log x)^{2/5}$. We sketch the older version of the algorithm in Appendix A.

Of course, we could use [5] as a black box to reduce space consumption in some of our routines, while leaving everything else as it is in the current version. Time complexity would increase slightly, while space complexity would be much reduced. More precisely: using [5] as a black box, and keeping everything else the same, we could compute *M*(*x*) in $O(x^{3/5} (\log x))$ word operations (and hence time $O(x^{3/5} (\log x)^2 \log \log x)$) and space $O(x^{1/5} (\log x)^{5/3})$. We choose to focus instead on the version of the algorithm reflected in the main theorem; it is faster but less space-efficient.

### Notation and algorithmic conventions

As usual, we write $f(x) = O(g(x))$ to denote that there is a positive constant *C* such that $|f(x)| \le C g(x)$ for all sufficiently large *x*. The notation $f(x)\ll g(x)$ is synonymous to $f(x)=O(g(x))$. We use $f(x) = O^*(g(x))$ to indicate something stronger, namely, $|f(x)| \le g(x)$ for all *x*.

For $x \in \mathbb {R}$, we write $\lfloor x \rfloor$ for the largest integer $\le x$, and $\{x\}$ for $x - \lfloor x \rfloor$. Thus, $\{x\} \in [0, 1)$ no matter whether $x < 0$, $x = 0$, or $x > 0$.

We write $\log _b x$ to mean the logarithm base *b* of *x*, *not* $\log \log \cdots \log x$ ($\log$ iterated *b* times).

We will count space in bits. We will assume that the time it takes to multiply two *n*-bit numbers ($n>1$) is $O(n \log n)$, as shown by [7]. (This is a more than reasonable assumption in practice, even if we use older algorithms. In all of our experiments, $n\le 128$; we could consider $n=196$ or $n=256$, but much larger *n* would correspond to values of *x* so large that an algorithm running in time $>x^{3/5}$ would not be practical.) We will also assume that accessing $O(\log x)$ consecutive bits in an array of length $\le x$ takes time $O(\log x)$.

All of the pseudocode for our algorithms appears at the end of this paper. We will keep track of the space and number of (word) operations used by each function. Total time (measured in bit operations) will be bounded by the number of word operations times $O(\log x \log \log x)$, since all of our arithmetic operations will be on integers of size $x^{O(1)}$ (or rationals of numerator and denominator bounded by $x^{O(1)}$), and all of our arrays will be of size much smaller than *x*. Since it may not be immediately clear that we cannot hope for a factor of $O(\log x)$ rather than $O(\log x \log \log x)$, we will point out two bottlenecks where the factor is indeed $O(\log x \log \log x)$. This is so because of multiplications, square-roots and divisions; addition and memory access only impose a factor of $O(\log x)$.

## Preparatory work: identities

We will start from the identity

2.1 $$\begin{aligned} \mu (n) = - \mathop {\sum _{m_1 m_2 n_1 = n}}_{m_1,m_2\le u} \mu (m_1) \mu (m_2) + {\left\{ \begin{array}{ll} 2 \mu (n) &{}\text {if }n\le u\\ 0 &{}\text {otherwise,} \end{array}\right. } \end{aligned}$$

valid for $n\le x$ and $u\ge \sqrt{x}$. (We will set $u=\sqrt{x}$.) This identity is simply the case $K=2$ of Heath-Brown’s identity for the Möbius function: for all $K \ge 1, n \ge 1$, and $u \ge n^{1/K}$,

$$\begin{aligned} \mu (n) = - \sum _{1 \le k \le K} (-1)^k \left( {\begin{array}{c}K\\ k\end{array}}\right) \mathop {\sum _{m_1 \ldots m_k n_1 \ldots n_{k-1}=n}}_{m_1,\ldots ,m_k\le u} \mu (m_1) \ldots \mu (m_k). \end{aligned}$$

(See [8, (13.38)]; note, however, that there is a typographical error under the sum there: $m_1\dotsc m_k n_1\dotsc n_{k}=n$ should be $m_1\dotsc m_k n_1\dotsc n_{k-1}=n$.) Alternatively, we can derive (2.1) immediately from Vaughan’s identity for $\mu$: that identity would, in general, have a term consisting of a sum over all decompositions $m_1 m_2 n_1 = n$ with $m_1,m_2>u$, but that term is empty because $u^2\ge x$.

We sum over all $n \le x$, and obtain

2.2 $$\begin{aligned} \begin{aligned} M(x) = 2 M(u) - \sum _{n\le x} \mathop {\sum _{m_1 m_2 n_1 = n}}_{m_1, m_2\le u} \mu (m_1) \mu (m_2) . \end{aligned} \end{aligned}$$

for $$u\ge \sqrt{x}$$.

Before we proceed, let us compare matters to the initial approach in [1]. Lemma 2.1 in [1] states that

2.3 $$\begin{aligned} M(x) = M(u) - \sum _{m \le u} \mu (m) \sum _{\frac{u}{m} < n \le \frac{x}{m}} M\left( \frac{x}{mn}\right) \end{aligned}$$

for $1\le u\le x$. This identity is due to Lehman [11, p. 314]; like Vaughan’s identity, it can be proved essentially by Möbius inversion. For $u=\sqrt{x}$, this identity is equivalent to (2.1), as we can see by a change of variables and, again, Möbius inversion.

We will set $u=\sqrt{x}$ once and for all. We can compute *M*(*u*) in (2.2) in $O(u \log \log u)$ operations and space $O(\sqrt{u})$, by a segmented sieve of Eratosthenes. (Alternatively, we can compute *M*(*u*) in $O(u \log u)$ operations and space $O(u^{1/3} (\log u)^{2/3})$, using the space-optimized version of the segmented sieve of Eratosthenes in [5].) Thus, we will be able to focus on the other term on the right side of (2.2). We can write, for any $v\le u$,

2.4 $$\begin{aligned} \begin{aligned} \sum _{n\le x} \mathop {\sum _{m_1 m_2 n_1 = n}}_{m_1, m_2\le u} \mu (m_1) \mu (m_2) =&\sum _{n\le x}\; \mathop {\sum _{m_1 m_2 n_1 = n}}_{m_1, m_2\le v} \mu (m_1) \mu (m_2)\\&+ \sum _{n\le x} \mathop {\mathop {\sum _{m_1 m_2 n_1 = n}}_{m_1, m_2\le u}}_{\max ( m_1,m_2)>v} \mu (m_1) \mu (m_2). \end{aligned}\end{aligned}$$

In this way, computing *M*(*x*) reduces to computing the two double sums on the right side of (2.4).

## The case of a large non-free variable

Let us work on the second sum in (2.4) first. It is not particularly difficult to deal with; there are a few alternative procedures that would lead to roughly the same number of operations, and several that would lead to a treatment for which the number of operations would be larger only by a factor of $\log x$.

Clearly,

3.1 $$\begin{aligned} \begin{aligned} \sum _{n\le x} \mathop {\mathop {\sum _{m_1 m_2 n_1 = n}}_{m_1, m_2\le u}}_{\max ( m_1,m_2)>v} \mu (m_1) \mu (m_2) =&\sum _{v<m\le u} \mu (m)^2 \left\lfloor \frac{x}{m^2}\right\rfloor \\&+ 2 \sum _{n\le x} \mathop {\mathop {\sum _{m_1 m_2 n_1 = n}}_{v< m_1\le u}}_{m_2<m_1} \mu (m_1) \mu (m_2) \end{aligned}\end{aligned}$$

and

3.2 $$\begin{aligned} \sum _{n\le x} \mathop {\mathop {\sum _{m_1 m_2 n_1 = n}}_{v< m_1\le u}}_{m_2<m_1} \mu (m_1) \mu (m_2) = \sum _{v<a\le u} \mu (a) \sum _{r\le \frac{x}{a}} \mathop {\sum _{b|r}}_{b<a} \mu (b).\end{aligned}$$

It is evident that the first sum on the right in (3.1) can be computed in $O(u \log \log u)$ operations and space $O(\sqrt{u})$, again by a segmented sieve. (Alternatively, we can compute it in space $O(u^{1/3} (\log u)^{2/3})$ and $O(u \log u)$ operations, using the segmented sieve in [5].)

Write $$D(r,y) = \sum _{b|r: b\le y} \mu (b)$$. Then

$$\begin{aligned} \begin{aligned} \sum _{r\le \frac{x}{a}} \mathop {\sum _{b|r}}_{b<a} \mu (b)&= \sum _{r\le \frac{x}{a}} \mathop {\sum _{b|r}}_{b\le \frac{x}{r}} \mu (b) - \sum _{r\le \frac{x}{a}} \mathop {\sum _{b|r}}_{a\le b\le \frac{x}{r}} \mu (b)\\&= \sum _{r\le \frac{x}{a}} D\left( r,\frac{x}{r}\right) - \sum _{b\ge a} \mu (b) \sum _{r\le \frac{x}{b}} 1 = S\left( \frac{x}{a}\right) - \sum _{b\ge a} \mu (b) \left\lfloor \frac{x}{b^2}\right\rfloor .\end{aligned} \end{aligned}$$

where $S(m) = \sum _{r\le m} D(r;x/r) = 1 + \sum _{x/u<r\le m} D(r;x/r)$, since $D(r;x/r) = \sum _{b|r: b\le x/r} \mu (b) = \sum _{b|r} \mu (r)$ for $r\le \sqrt{x} = u$.

Thus, to compute the right side of (3.2), it makes sense to let *n* take the values $\lfloor u\rfloor , \lfloor u\rfloor -1,\dotsc , \lfloor v\rfloor +1$ in descending order; as *n* decreases, *x*/*n* increases, and we compute *D*(*r*; *x*/*r*), and thus *S*(*x*/*n*), for increasing values of *r*. Computing all values of $\mu (a)$ for $v<a\le u$ using a segmented sieve of Eratosthenes takes $O(u\log \log u)$ operations and space $O(\sqrt{u})$.

The main question is how to compute *D*(*r*; *x*/*r*) efficiently for all *r* in a given segment. Using a segmented sieve of Eratosthenes, we can determine the set of prime divisors of all *r* in an interval of the form $[y, y+\Delta ]$, $|\Delta |\ge \sqrt{y}$, in $O(\Delta \log \log y)$ operations and space $O(\Delta \log y)$. We want to compute the sum $D(r;x/r) = \sum _{b|r: b<x/r} \mu (b)$ for all *r* in that interval. The naive approach would be to go over all divisors *b* of all integers *r* in $[y, y+\Delta ]$; since those integers have $\log y$ divisors on average, doing so would take $O(\Delta \log y)$ operations. Fortunately, there is a less obvious way to compute *D*(*r*; *x*/*r*) using, on average, $O(\log \log y)$ operations. We will need a simple lemma on the anatomy of integers.

### Lemma 3.1

Let $P_z(n) = \prod _{p \le z: p \mid n} p.$ For *z*, *N*, *a* arbitrary and $N < n \le 2N$ random, the expected value of

3.3 $$\begin{aligned} \mathop {\sum _{\frac{a}{P_z(n)}< d \le 2a}}_{p \mid d \Rightarrow p > z}  \sum _{\begin{array}{c} d' \mid n:\; d'\text { squarefree} \\ p \mid d' \Rightarrow z^{1/2} < p \le z \end{array}} 1 \end{aligned}$$

is *O*(1).

### Proof

For any fixed positive integer *K*, the numbers $N<n \le 2 N$ with $P_z(n) = K$ are of the form $m \cdot \prod _{p \le z: p \mid n} = m \cdot K,$ where *m* can be any of the *z*-rough integers $N/K <m \le 2 N/K$. Let us consider how many divisors *d*|*m* with properties with $p \mid d \Rightarrow p > z$ and $\frac{a}{P_z(n)} < d \le 2a$ there are on average as *m* varies on (*N*/*K*, 2*N*/*K*].

We can assume that $z\le N/K$, as otherwise *m* has at most 2 divisors *d* free of prime factors $\le z$ (namely, $d=1$ and $d=m$). Then a random integer $m \in (N/K, 2N/K]$ with no prime factors $\le z$ has the following expected number of divisors in $(\frac{a}{K}, 2a]$:

$$\begin{aligned} \frac{1}{(N/K)/\log z} O\left( \sum _{\begin{array}{c} \frac{a}{K}< d \le 2a \\ p \mid d \Rightarrow p> z \end{array}} \frac{(N/K)/d}{\log z}\right) + O(1) = O\Big (1 + \sum _{\begin{array}{c} \frac{a}{K} < d \le 2a \\ p \mid d \Rightarrow p > z \end{array}} \frac{1}{d}\Big ) , \end{aligned}$$

since the number of integers in (*M*, 2*M*] with no prime factors up to *z* is $\gg M/\log z$ for $z\le M$ and $\ll M/\log z$ for $z>1$ and $M\ge 1$. (The term *O*(1) is there to account for $d=m$; in that case and only then, $(N/K)/d < 1$.)

Applying an upper bound sieve followed by partial summation, we see that

$$\begin{aligned} \sum _{\begin{array}{c} \frac{a}{K} < d \le 2a \\ p \mid d \Rightarrow p> z \end{array}} \frac{1}{d} \ll (\log 2a - \log a/K) \prod _{p \le z} \left( 1 - \frac{1}{p}\right) + 1. \end{aligned}$$

(The term *O*(1) comes from $\sum _{a/K<d\le z a/K} 1/d$.) By Mertens’ Theorem, the product is $\ll 1/\log z$. Hence,

$$\begin{aligned} \sum _{\begin{array}{c} \frac{a}{K} < d \le 2a \\ e \mid d \Rightarrow e > z \end{array}} \frac{1}{d} = O\left( \frac{\log 2 a - \log a/K}{\log z} + 1\right) = O\left( \frac{\log 2 K}{\log z} + 1\right) . \end{aligned}$$

The number of divisors $d'|n$ with $p|d'\Rightarrow z^{1/2}<p\le z$ depends only on $K = P_z(n)$. Therefore, the expected value of (3.3) is

3.4 $$\begin{aligned} O\left( \mathbb {E}\left( \left( \frac{\log 2 P_z(n)}{\log z} + 1\right) \sum _{\begin{array}{c} d' \mid n:\; d'\text { squarefree} \\ p \mid d' \Rightarrow z^{1/2} < p \le z \end{array}} 1 \right) \right) .\end{aligned}$$

Now, $\log P_z(n) = \sum _{p|n} \log p$. Let $\xi$ denote the random variable given by

$$\begin{aligned} \xi = \sum _{\begin{array}{c} d' \mid n:\; d' \text { squarefree} \\ p \mid d' \Rightarrow z^{1/2} < p \le z \end{array}} 1 \end{aligned}$$

and let $A_p$ denote the event that $p \mid n$. Then (3.4) is at most a constant times

3.5 $$\begin{aligned} \mathbb {E}\Big ( \xi \Big ) + \frac{1}{\log z} \sum _{p\le z} \frac{\log p}{p} \mathbb {E}\Big ( \xi \Big |\; A_p \Big ).\end{aligned}$$

Clearly

$$\begin{aligned} \begin{aligned}\mathbb {E}\left( \xi \right)&\le \frac{1}{N} \sum _{n \le 2N} \sum _{\begin{array}{c} d' \mid n:\; d'\text { squarefree} \\ p \mid d' \Rightarrow z^{1/2}< p \le z \end{array}} 1 \ll \frac{1}{N} \sum _{\begin{array}{c} d\text { square-free} \\ p \mid d \Rightarrow z^{1/2}< p \le z \end{array}} \frac{N}{d}\\ {}&= \sum _{\begin{array}{c} d\text { square-free} \\ p \mid d \Rightarrow z^{1/2}< p \le z \end{array}} \frac{1}{d} = \prod _{z^{1/2} < p \le z}\left( 1 + \frac{1}{p}\right) \sim \frac{\log z}{\log z^{1/2}} \ll 1. \end{aligned} \end{aligned}$$

We must also estimate the conditional expectation: for $p\le z\le N$,

$$\begin{aligned} \begin{aligned} \mathbb {E}\Big (\xi \Big |\; A_p \Big )&\ll \frac{1}{N/p} \mathop {\sum _{n \le 2N}}_{p|n} \sum _{\begin{array}{c} d' \mid n:\; d'\text { squarefree} \\ p' \mid d' \Rightarrow z^{1/2}< p' \le z \end{array}} 1\\&\ll \frac{1}{N/p} \left( \sum _{\begin{array}{c} d\text { square-free}: p\not \mid d \\ p' \mid d \Rightarrow z^{1/2}< p' \le z \end{array}} \frac{N/p}{d} + \sum _{\begin{array}{c} d\text { square-free}: p|d \\ p' \mid d \Rightarrow z^{1/2}< p' \le z \end{array}} \frac{N/p}{d/p}\right) \\ {}&\ll \sum _{\begin{array}{c} d\text { square-free}: p\not \mid d \\ p' \mid d \Rightarrow z^{1/2}< p' \le z \end{array}} \frac{1}{d} \le \prod _{z^{1/2} < p \le z}\left( 1 + \frac{1}{p}\right) \ll 1. \end{aligned} \end{aligned}$$

Hence, the expression in (3.5) is

$$\begin{aligned} \ll 1 + \frac{1}{\log z} \sum _{p\le z} \frac{\log p}{p} \ll 1 + \frac{\log z}{\log z} \ll 1. \end{aligned}$$

$$\square $$

### Proposition 3.2

Define $D(n;a) = \sum _{d|n: d\le a} \mu (d)$. Let $N,A\ge 1$. For each $N<n\le 2 N$, let $A \le a(n) \le 2 A$. Then, given the factorization $n= p_1^{\alpha _1} p_2^{\alpha _2} \cdots p_r^{\alpha _r}$, where $p_1<p_2<\dotsc <p_r$, Algorithm 23 computes *D*(*n*; *a*(*n*)). in a number of operations that is $O(\log \log N)$ on average over $n=N+1,\dotsc , 2 N$.

### Proof

Algorithm 23 computes *D*(*n*; *a*) recursively: it calls itself to compute $D(n_0;a)$ and $D(n_0;a/p_r)$, where $n_0 = p_1 p_2 \cdots p_{r-1}$, and then returns $D(n;a) = D(n_0;a) - D(n_0;a/p_r)$. The contribution of $D(n_0;a)$ is that of divisors $\ell |n$ with $p_r\not \mid \ell$, whereas the contribution of $D(n_0;a/p_r)$ corresponds to that of divisors $\ell |n$ with $p_r|\ell$.

The algorithm terminates in any of three circumstances:

1. for $$a<1$$, returning $$D(n;a)=0$$,
2. for $n=1$ and $a\ge 1$, returning $D(n;a)=1$,
3. for $n>1$ and $a\ge n$, returning $D(n;a)=0$.

Here it is evident that the algorithm gives the correct output for the cases (1)–(2), whereas case (3) follows from $D(n;a) = \sum _{d|n: d\le a} \mu (d) = \sum _{d|n}\mu (d) = 0$ for $n>1$, $a\ge n$.

We can see recursion as traversing a *recursion tree*, with leaves corresponding to cases in which the algorithm terminates. (In the study of algorithms, trees are conventionally drawn with the root at the top.) The total number of operations is proportional to the number of nodes in the tree. If the algorithm were written to terminate only for $n=1$, the tree would have $2^r$ leaves; as it is, the algorithm is written so that some branches terminate long before reaching depth *r*. We are to bound the average number of nodes of the recursion tree for inputs $N<n\le 2 N$ and $a=a(n) \in [A,2A]$.

Say we are at the depth reached after taking care of all $p_i$ with $p_i>z$. The branches that have survived correspond to *d*|*n* with $p|d \Rightarrow p>z$, $d\le 2 A$ and $d>A/P_z(n)$. We are to compute $D(P_z(n);a/d)$. (If $d>2 A$, then $a/d<1$, and so our branch has terminated by case (1) above. If $d\le A/P_z(n)$, then $a/d\ge P_z(n)$, and we are in case (3).)

Now we continue running the algorithm until we take care of all $p_i$ with $p_i>z^{1/2}$. On each branch that survived up to depth $p>z$, the nodes between that depth and depth $p>z^{1/2}$ correspond to square-free divisors $d'|n$ such that $p|d\Rightarrow z^{1/2}<p\le z$.

By Lemma 3.1, we conclude that the average number of nodes in the tree corresponding to $z^{1/2}<p\le z$ is *O*(1). Letting $z = N, N^{1/2}, N^{1/4}, N^{1/8},\dotsc$, we obtain our result. $\square$

In this way, letting $\Delta = \sqrt{x/v}$, we can compute *D*(*r*; *x*/*r*) for all $x/u<r\le x/v$ in $O((x/v) \log \log (x/v))$ operations and space $O(\sqrt{x/v} \log (x/v))$. Summing values of *D*(*r*; *x*/*r*) for successive values of *r* to compute $S(m) = \sum _{r\le m} D(r;x/r)$ for $x/u<m\le x/v$ takes *O*(*x*/*v*) operations and additional space^1^*O*(1). As *a* decreases and $m=x/a$ increases, we may (and should) discard values of *S*(*m*) and *D*(*r*; *x*/*r*) that we no longer need, so as to keep space usage down.

We have thus shown that we can compute the right side of (3.2) in $O((x/v) \log \log x)$ operations and space $O(\sqrt{x/v}\cdot \log x)$ for any $1\le v\le u = \sqrt{x}$.

It is easy to see that, if we use the algorithm in [5, Main Thm.] instead of the classical segmented sieve of Eratosthenes, we can accomplish the same task in $O((x/v) \log x)$ operations and space $O((x/v)^{1/3} (\log x)^{5/3})$.

**Bitwise time bottleneck.** Since our operations are all on integers $\le x$, each of our (word) operations involves $O(\log x \log \log x)$ bit operations, and so it is clear that our $O((x/v) \log \log x)$ operations take at most

$$\begin{aligned} O((x/v) \log x (\log \log x)^2) \end{aligned}$$

bit operations. The question is whether one could do a little better.

The segmented sieve of Eratosthenes for factorization takes only

$$\begin{aligned} O((x/v) \log x \log \log x) \end{aligned}$$

bit operations. (In the final step, multiply small factors before large ones.) However, our procedure for computing *D*(*n*; *a*(*n*)) does take time proportional to $(x/v) \log x (\log \log x)^2$ in total. The reason is the following. Recall that, to keep the number of operations low, Algorithm 23 uses (and multiplies integers by) large primes before small ones. For *a* a fixed power of *N*, a positive proportion of integers $n\asymp N$ have prime factors between $a^{1/3}$ and $a^{2/3}$ (say). Those prime factors are found early on; they correspond to the first two or three levels of the recursion tree in the proof of Prop. 3.2. Then every node further down in the recursion tree involves a multiplication by a number of size at least $a^{1/3}$. That multiplication takes $\gg \log a^{1/3} \log \log a^{1/3} \gg \log N \log \log N$ bit operations. Here $\log N\gg \log x$. Thus, in our current algorithm, the number of bit operations is, in fact, on the order of $(x/v) \log x (\log \log x)^2$.

**A few words on the implementation.** See Algorithm 2.

*Choice of* $\Delta$. The size of the segments used by the sieve is to be chosen at the outset: $\Delta = C \max (\sqrt{u},\sqrt{x/v}) = C \sqrt{x/v}$ (for some choice of constant $C\ge 1$) if we use the classical segmented sieve (SegFactor), or

3.6 $$\begin{aligned} \Delta = C \max \left( \root 3 \of {u} (\log u)^{2/3}, \root 3 \of {\frac{x}{v}} (\log x/v)^{2/3}\right) = C \root 3 \of {\frac{x}{v}} \left( \log \frac{x}{v}\right) ^{2/3} \end{aligned}$$

for the improved segmented sieve in [5, Main Thm.].

*Memory usage.* It is understood that calls such as $F\leftarrow \textsc {SegFactor}(a_0,\Delta )$ will result in freeing or reusing the memory previously occupied by *F*. (In other words, “garbage-collection” will be taken care of by either the programmer or the language.)

*Parallelization.* Most of the running time is spent in function SArr (Algorithm 4), which is easy to parallelize. We can let each processor sieve a block of length $\Delta$. Other than that – the issue of computing an array of sums $\textbf{S}$ (as in Algorithm 4) in parallel is a well-known problem (*prefix sums*), for which solutions of varying practical efficiency are known. We follow a common two-level algorithm: first, we divide the array into as many blocks as there are processing elements; then (level 1) we let each processing element compute, in parallel, an array of prefix sums for each block, ending with the total of the block’s entries; then we compute prefix sums of these totals to create offsets; finally (level 2), we let each processing element add its block’s offset to all elements of its block.

## The case of a large free variable

We now show how to compute the first double sum on the righthand side of (2.4). That double sum equals

4.1 $$\begin{aligned} \sum _{m, n \le v} \mu (m) \mu (n) \left\lfloor \frac{x}{mn} \right\rfloor .\end{aligned}$$

Note that, in [1], this turns out to be the easy case. However, they take $v = x^{1/3}$, while we will take $v = x^{2/5}$. As a result, we have to take much greater care with the computation to ensure that the runtime does not become too large.

### A first try

We begin by splitting $$[1, v] \times [1, v]$$ into neighborhoods *U* around points $$(m_0, n_0)$$. For simplicity, we will take these neighborhoods to be rectangles of the form $$I_x \times I_y$$ with $$I_x = [m_0 - a, m_0 + a)$$ and $$I_y = [n_0 - b, n_0 + b)$$, where $$\sqrt{m_0}\ll a < m_0$$ and $$\sqrt{n_0}\ll b < n_0$$. (In Sect. 5, we will partition the two intervals [1, *v*] into intervals of the form $$[x_0, (1+\eta )x_0)$$ and $$[y_0, (1+\eta )y_0)$$, with $$0< \eta \le 1$$ a constant. We will then specify *a* and *b* for given $$x_0$$ and $$y_0$$, and subdivide $$[x_0, (1+\eta )x_0) \times [y_0, (1+\eta )y_0)$$ into rectangles $$I_x \times I_y$$ with $$|I_x| = 2 a$$ and $$|I_y| = 2 b$$.) Applying a local linear approximation to the function $$\frac{x}{mn}$$ on each neighborhood yields

4.2 $$\begin{aligned} \frac{x}{mn} = \frac{x}{m_0 n_0} + c_x(m - m_0) + c_y (n - n_0) + \textrm{ET}_{\textrm{quad}}(m,n),\end{aligned}$$

where $\textrm{ET}_{\textrm{quad}}(m,n)$ is a quadratic error term (that is, a term whose size is bounded by $O(\max (n-n_0, m-m_0)^2)$ and

$$c_x = \frac{-x}{m_0^2 n_0}, \ c_y = \frac{-x}{m_0 n_0^2}.$$

The quadratic error term will be small provided that *U* is small. We will show how to choose *U* optimally at the end of this section. The point of applying the linear approximation is that it will ultimately allow us to separate the variables in our sum. The one complicating factor is the presence of the floor function. If we temporarily ignore both the floor function in (4.1) and the quadratic error term, we can see very clearly how the linear approximation helps us. To wit:

4.3 $$\begin{aligned} \sum _{(m, n) \in I_x \times I_y} \mu (m) \mu (n) \frac{x}{mn} \end{aligned}$$

is approximately equal to

4.4 $$\begin{aligned}&\sum _{(m, n) \in I_x \times I_y} \mu (m)\mu (n) \left( \frac{x}{m_0 n_0} + c_x(m - m_0) + c_y(n - n_0)\right) \nonumber \\&\quad = \left( \sum _{m \in I_x} \mu (m) \left( \frac{x}{m_0 n_0} + c_x(m - m_0)\right) \right) \cdot \sum _{n \in I_y} \mu (n) \nonumber \\&\qquad + \left( \sum _{n \in I_y} \mu (n) c_y (n - n_0)\right) \cdot \sum _{m \in I_x} \mu (m). \end{aligned}$$

One can use the segmented sieve of Eratosthenes to compute the values of $\mu (m)$ for $m \in I_x$ and $\mu (n)$ for $n \in I_y$. If $a < \sqrt{x_0}$ or $b < \sqrt{y_0}$, we compute the values of $\mu$ in segments of length about $\sqrt{x_0}$ or $\sqrt{y_0}$ and use them for several neighborhoods $I_x \times I_y$. In any event, computing 4.4 given $\mu (m)$ for $m \in I_x$ and $\mu (n)$ for $n \in I_y$ takes only $O(\max (a, b))$ operations and negligible space.

### Handling the difference between reality and an approximation

Proceeding as above, we can compute the sum

$$\begin{aligned} S_0:= \sum _{(m, n) \in I_x \times I_y} \mu (m) \mu (n) \left( \left\lfloor \frac{x}{m_0 n_0} + c_x(m - m_0)\right\rfloor + \left\lfloor c_y(n - n_0)\right\rfloor \right) \end{aligned}$$

in $O(\max (a,b))$ operations and space $O(\log \max (x_0,y_0))$, given arrays with the values of $\mu (m)$ and $\mu (n)$. The issue is that $S_0$ is not the same as

$$\begin{aligned} S_1 := \sum _{(m, n) \in I_x \times I_y} \mu (m) \mu (n) \left( \left\lfloor \frac{x}{m_0 n_0} + c_x(m - m_0) + c_y(n - n_0)\right\rfloor \right) ,\end{aligned}$$

and it is certainly not the same as the sum we actually want to compute, namely,

$$\begin{aligned} S_2 := \sum _{(m, n) \in I_x \times I_y} \mu (m) \mu (n) \left\lfloor \frac{x}{m n}\right\rfloor . \end{aligned}$$

From now on, we will write

$$\begin{aligned} L_0(m,n)= & {} \left\lfloor \frac{x}{m_0 n_0} + c_x(m - m_0)\right\rfloor + \left\lfloor c_y(n - n_0)\right\rfloor ,\\ L_1(m,n)= & {} \left\lfloor \frac{x}{m_0 n_0} + c_x(m - m_0) + c_y(n - n_0)\right\rfloor ,\;\;\;\;\; L_2(m,n) = \left\lfloor \frac{x}{m n}\right\rfloor . \end{aligned}$$

Here $m_0$, $n_0$ and *x* are understood to be fixed. Our challenge will be to show that the weights $L_2-L_1$ and $L_1-L_0$ actually have a simple form – simple enough that $S_2-S_1$ and $S_1-S_0$ can be computed quickly.

We approximate $c_y$ by a rational number $a_0/q$ with $q \le Q = 2b$ such that

$$\begin{aligned} \delta := c_y - a_0/q \end{aligned}$$

satisfies $$|\delta | \le 1/q Q.$$ Thus,

4.5 $$\begin{aligned} \left| c_y (n-n_0) - \frac{a_0 (n-n_0)}{q}\right| \le \frac{1}{2 q} .\end{aligned}$$

We can find such an $\frac{a_0}{q}$ in $O(\log Q)$ operations using continued fractions (see Algorithm 9).

Write $r_0 = r_0(m)$ for the integer such that the absolute value of

4.6 $$\begin{aligned} \beta = \beta _m := \left\{ \frac{x}{m_0 n_0} + c_x (m-m_0)\right\} - \frac{r_0}{q} \end{aligned}$$

is minimal (and hence $\le 1/2 q$). If there are two such values, choose the greater one. Then

4.7 $$\begin{aligned} - \frac{1}{2 q} \le \beta < \frac{1}{2 q} .\end{aligned}$$

We will later make sure that we choose our neighborhoods $I_x\times I_y$ so that $|\textrm{ET}_{\textrm{quad}}(m,n)|\le 1/2 b$, where $\textrm{ET}_{\textrm{quad}}(m,n)$ is defined by (4.2). We also know that $\textrm{ET}_{\textrm{quad}}(m,n)>0$, since the function $(m,n)\mapsto x/m n$ is convex. We are of course assuming that $I_x\times I_y$ is contained in the first quadrant, and so $(m,n)\mapsto x/m n$ is well-defined on it.

The aforementioned notation will be used throughout this section.

#### Lemma 4.1

Let $$(m,n)\in I_x\times I_y$$. Unless $$a_0(n - n_0) + r_0 \in \{0, -1\} {{\,\mathrm{\,mod}\,}}q$$,

$$\begin{aligned} L_2(m,n) = L_1(m,n). \end{aligned}$$

#### Proof

Since $0 < \textrm{ET}_{\textrm{quad}}(m, n) \le 1/2b$, we can have

4.8 $$\begin{aligned} \left\lfloor \frac{x}{m n}\right\rfloor \ne \left\lfloor \frac{x}{m_0 n_0} + c_x(m - m_0) + c_y(n - n_0)\right\rfloor \end{aligned}$$

(in which case the left side equals the right side plus 1) only if

4.9 $$\begin{aligned} \left\{ \frac{x}{m_0 n_0} + c_x(m - m_0) + c_y(n - n_0)\right\} \ge 1 - \frac{1}{2 b}.\end{aligned}$$

Since $$q\le 2 b$$ and

$$\begin{aligned} \frac{x}{m_0 n_0} + c_x(m - m_0) + c_y(n - n_0) \in \frac{a_0 (n-n_0) + r_0}{q} + \left[ -\frac{1}{q},\frac{1}{q}\right) , \end{aligned}$$

we see that (4.9) can be the case only if $a_0 (n-n_0) + r_0$ is in $\{0,-1\} {{\,\mathrm{\,mod}\,}}q$. $\square$

#### Lemma 4.2

Let $$(m,n)\in I_x\times I_y$$. Unless $$a_0(n - n_0) + r_0 \equiv 0 \pmod {q}$$,

4.10 $$\begin{aligned} L_1(m,n) - L_0(m,n) =&{\left\{ \begin{array}{ll} 0 &{} \text {if }r_0 + \overline{a_0 (n-n_0)} \le q,\\ 1 &{} \text {otherwise,} \end{array}\right. }\end{aligned}$$

4.11 $$\begin{aligned}&+ {\left\{ \begin{array}{ll} 1 &{} \text {if }q|(n-n_0) \wedge (\delta (n-n_0)< 0),\\ 0 &{} \text {otherwise,} \end{array}\right. } \end{aligned}$$

where $\overline{a}$ denotes the integer in $\{0,1,\dotsc ,q-1\}$ congruent to *a* modulo *q*.

#### Proof

Recall that, for all real numbers *A* and *B*,

$$\begin{aligned} \lfloor A + B \rfloor - (\lfloor A \rfloor + \lfloor B \rfloor ) = {\left\{ \begin{array}{ll} 0, &{} \textrm{if} \ \{A\} + \{B\} < 1 \\ 1, &{} \mathrm {otherwise.} \end{array}\right. } \end{aligned}$$

Thus, $L_1(m,n)-L_0(m,n)$ is either 0 or 1, and it is 1 if and only if

4.12 $$\begin{aligned} \left\{ \frac{x}{m_0 n_0} + c_x(m - m_0)\right\} + \left\{ c_y(n - n_0)\right\} \end{aligned}$$

is $\ge 1$. By (4.5) and (4.7), the quantity in (4.12) lies in

$$\begin{aligned} \frac{r_0}{q} + \left\{ \frac{a_0 (n-n_0)}{q}\right\} + \left[ -\frac{1}{q}, \frac{1}{q}\right) \end{aligned}$$

unless, possibly, if $a_0 (n-n_0)\equiv 0 {{\,\mathrm{\,mod}\,}}q$, that is, if $q|(n-n_0)$. Hence, unless $a_0 (n- n_0) + r_0 \equiv 0 {{\,\mathrm{\,mod}\,}}q$ or $q|(n-n_0)$, the expression in (4.12) is $\ge 1$ if and only if $r_0/q + \{a_0 (n-n_0)/q\} \ge 1$. Moreover, if $q|(n-n_0)$ but $a_0 (n- n_0) + r_0 \not \equiv 0 {{\,\mathrm{\,mod}\,}}q$, it is easy to see that the expression in (4.12) is $<1$ iff $\delta (n-n_0) = c_y (n-n_0) - a_0 (n-n_0)/q$ is $\ge 0$. $\square$

It follows immediately from Lemmas 4.1 and 4.2 that

4.13 $$\begin{aligned} L_2(m,n)-L_0(m,n) = {\left\{ \begin{array}{ll} 0 &{} \text {if }r_0 + \overline{a_0 (n-n_0)} \le q,\\ 1 &{} \text {otherwise,} \end{array}\right. } \end{aligned}$$

unless $$r_0 + a_0 (n-n_0) \in \{0,-1\} {{\,\mathrm{\,mod}\,}}q$$.

Note that the first term on the right side of (4.13) depends only on $n {{\,\mathrm{\,mod}\,}}q$ (and $a_0 {{\,\mathrm{\,mod}\,}}q$ and $r_0$), and the second term depends only on $n{{\,\mathrm{\,mod}\,}}q$, ${{\,\textrm{sgn}\,}}(n-n_0)$ and ${{\,\textrm{sgn}\,}}(\delta )$ (and not on $r_0$; hence it is independent of *m*). Given the values of $\mu (n)$ for $n\in I_y$, it is easy to make a table of

$$\begin{aligned} \rho _r = \mathop {\sum _{n\in I_y}}_{a_0 (n-n_0)\equiv r {{\,\mathrm{\,mod}\,}}q} \mu (n) \end{aligned}$$

for $r\in \mathbb {Z}/q \mathbb {Z}$ in *O*(*b*) operations and space $O(q \log b)$, and then a table of

$$\begin{aligned} \sigma _r = \mathop {\sum _{n\in I_y}}_{\overline{a_0 (n-n_0)} > q- r} \mu (n) \end{aligned}$$

for $0\le r\le q$ in *O*(*q*) operations and space $O(q \log b)$. We also compute

$$\begin{aligned} \mathop {\mathop {\sum _{n\in I_y}}_{q|n-n_0}}_{\delta \cdot (n-n_0) < 0} \mu (n) \end{aligned}$$

once and for all. It remains to deal with the problematic cases $a_0 (n-n_0)+r_0\in \{0,-1\} {{\,\mathrm{\,mod}\,}}q$.

#### Lemma 4.3

Let $(m,n)\in I_x\times I_y$. If $a_0(n - n_0) + r_0 \equiv -1 \pmod {q}$ and $q>1$, then

$$\begin{aligned} L_2(m,n) - L_1(m,n) = {\left\{ \begin{array}{ll} 1 &{} \text {if }n \not \in I,\\ 0 &{} \text {if }n \in I, \end{array}\right. } \end{aligned}$$

where $I = (\textbf{x}_-,\textbf{x}_+)$ if the equation

$$\begin{aligned} \gamma _2 \textbf{x}^2 + \gamma _1 \textbf{x} + \gamma _0 = 0 \end{aligned}$$

has real roots $\textbf{x}_-< \textbf{x}_+$, and $I=\emptyset$ otherwise. Here $\gamma _0 = x q$, $\gamma _2 = - a_0 m$ and

$$\begin{aligned} \gamma _1&= \left( -\left\lfloor \frac{x}{m_0 n_0} + c_x(m - m_0)\right\rfloor q - (r_0+1) + a_0 n_0\right) m . \end{aligned}$$

#### Proof

The question is whether $L_2(m,n)>L_1(m,n)$. Since

4.14 $$\begin{aligned} -1/2q \le \beta <1/2 q \ {\textrm{and}} \ |\delta (n-n_0)|\le 1/2 q,\end{aligned}$$

we know that

$$\begin{aligned}&\left\{ \frac{x}{m_0 n_0} + c_x(m - m_0) + c_y(n - n_0)\right\} = \left\{ \frac{r_0}{q} + \beta + \frac{a_0 (n-n_0)}{q} + \delta (n-n_0)\right\} \\&\quad = \left\{ - \frac{1}{q} + \beta + \delta (n-n_0)\right\} = \frac{q-1}{q} + \beta + \delta (n-n_0), \end{aligned}$$

where the last line follows from (4.14). Hence, $L_2(m,n)>L_1(m,n)$ if and only if

4.15 $$\begin{aligned} \frac{x}{m n} - \left( \frac{x}{m_0 n_0} + c_x(m - m_0) + c_y(n - n_0)\right) \ge \frac{1}{q} - \beta - \delta (n-n_0).\end{aligned}$$

This, in turn, is equivalent to

4.16 $$\begin{aligned} \frac{c_0}{n} + c_1 + c_2 n \ge 0,\end{aligned}$$

where $$c_0 = x/m$$, $$c_2 = -a_0/q$$ and

$$\begin{aligned} c_1&= -\left( \frac{x}{m_0 n_0} + c_x(m - m_0)-\beta \right) + \frac{a_0}{q} n_0 - \frac{1}{q} \\ {}&= -\left\lfloor \frac{x}{m_0 n_0} + c_x(m - m_0)\right\rfloor - \frac{r_0+1}{q} + \frac{a_0}{q} n_0 .\end{aligned}$$

Since $a_0/q$ is a Diophantine approximation to $c_y = - x/m_0 n_0^2 < 0$, it is clear that $a_0/q$ is non-positive. Consequently, if $q>1$, $a_0$ must be negative, since $a_0$ and *q* are coprime. Hence, $c_2$ is positive, and so (4.16) holds iff $n\not \in I$, where $I = (\textbf{x}_-,\textbf{x}_+)$ if the equation

$$\begin{aligned} c_2 \textbf{x}^2 + c_1 \textbf{x} + c_0 = 0 \end{aligned}$$

has real roots $\textbf{x}_-\le \textbf{x}_+$, and $I=\emptyset$ otherwise. $\square$

Solving a quadratic equation is not computationally expensive; in practice, the function $n\mapsto \lfloor \sqrt{n}\rfloor$ generally takes roughly as much time to compute as a division. Thus it makes sense to count $x\mapsto \lfloor \sqrt{n}\rfloor$ as one (word) operation, like the four basic operations $+$, −, $\cdot$, /. Computing $\lfloor \sqrt{n}\rfloor$ takes $O(\log n \log \log n)$ bit operations, just like multiplication and division.

What we have to do now is keep a table of

$$\begin{aligned} \rho _{r,\le n'} = \mathop {\sum _{n\in I_y, n\le n'}}_{a_0 (n-n_0)\equiv r {{\,\mathrm{\,mod}\,}}q} \mu (n). \end{aligned}$$

We need only consider values of $n'$ satisfying $a_0 (n' - n_0)\equiv r {{\,\mathrm{\,mod}\,}}q$ (since $\rho _{r,\le n'} = \rho _{r,\le n''}$ for $n''$ the largest number $n''\le n'$ with $a_0 (n'' - n_0)\equiv r {{\,\mathrm{\,mod}\,}}q$). It is then easy to see that we can construct the table in *O*(*b*) operations and space $O(b \log b)$, simply letting *n* traverse $I_y$ from left to right. (In the end, we obtain $\rho _r$ for every $r\in \mathbb {Z}/ q\mathbb {Z}$.) In the remaining lemmas, we show how to handle the cases where $a_0(n - n_0) + r_0 \equiv 0 \pmod {q}$.

#### Lemma 4.4

Let $(m,n)\in I_x\times I_y$. If $a_0(n - n_0) + r_0 \equiv 0 \pmod {q}$, then

$$\begin{aligned} L_1(m,n) - L_0(m,n) = {\left\{ \begin{array}{ll} 0 &{} \text {if }n \not \in I,\\ 1 &{} \text {if }n \in I, \end{array}\right. } \end{aligned}$$

where, if $$r_0\not \equiv 0 {{\,\mathrm{\,mod}\,}}q$$,

$$\begin{aligned} I = {\left\{ \begin{array}{ll} n_0 - \frac{\beta }{\delta } + \frac{1}{\delta } \cdot [0,\infty ) &{}\text {if }\delta \ne 0,\\ \mathbb {R} &{} \text {if }\delta =0\text { and }\beta \ge 0,\\ \emptyset &{} \text {if }\delta =0\text { and }\beta < 0,\end{array}\right. } \end{aligned}$$

and, if $$r_0\equiv 0 {{\,\mathrm{\,mod}\,}}q$$,

$$\begin{aligned} I = {\left\{ \begin{array}{ll} \mathbb {R} &{}\text {if }\beta<0\text { and }\delta<0\\ (-\infty ,n_0] \cup [n_0 - \frac{\beta }{\delta },\infty ) &{}\text {if }\beta <0\text { and }\delta>0\\ n_0 + \frac{1}{\delta } [-\beta ,0) &{}\text {if }\beta >0\text { and }\delta \ne 0,\\ \emptyset &{} \text {otherwise.}\end{array}\right. } \end{aligned}$$

#### Proof

Since $$\{a_0 (n-n_0)/q\} = \{-r_0/q\}$$,

$$\begin{aligned} \left\{ \frac{x}{m_0 n_0} + c_x(m - m_0)\right\} + \left\{ c_y(n - n_0)\right\}&= \left\{ \frac{r_0}{q} + \beta \right\} + \left\{ -\frac{r_0}{q} + \delta (n- n_0)\right\} . \end{aligned}$$

Recall that $-1/2q\le \beta <1/2 q$ and $|\delta (n-n_0)|\le 1/2 q$. For $r_0\not \equiv 0 {{\,\mathrm{\,mod}\,}}q$, $\{r_0/q+\beta \}+\{-r_0/q + \delta (n-n_0)\}\ge 1$ iff $\beta +\delta (n-n_0)\ge 0$. We treat the case $r_0 \equiv 0 {{\,\mathrm{\,mod}\,}}q$ separately: $\{\beta \}+\{\delta (n-n_0)\}\ge 1$ iff either (a) $\beta <0$ and $\delta (n-n_0)<0$, or (b) $\beta \delta (n-n_0) < 0$ and $\beta +\delta (n-n_0)\ge 0$. $\square$

#### Lemma 4.5

Let $(m,n)\in I_x\times I_y$. If $a_0(n - n_0) + r_0 \equiv 0 \pmod {q}$ and $q>1$,

$$\begin{aligned} L_2(m,n) - L_1(m,n) = {\left\{ \begin{array}{ll} 0 &{} \text {if }n \not \in I\cap J,\\ 1 &{} \text {if }n \in I\cap J, \end{array}\right. } \end{aligned}$$

where $I = [\textbf{x}_-,\textbf{x}_+]$ if the equation

$$\begin{aligned} \gamma _2 \textbf{x}^2 + \gamma _1 \textbf{x} + \gamma _0 = 0 \end{aligned}$$

has real roots $\textbf{x}_-\le \textbf{x}_+$, and $I=\emptyset$ otherwise, whereas $J = n_0 -\beta /\delta -\frac{1}{\delta } (0,\infty )$ if $\delta \ne 0$, $J= \emptyset$ if $\delta =0$ and $\beta \ge 0$ and $J= (-\infty ,\infty )$ if $\delta =0$ and $\beta <0$. Here $\gamma _0 = x q$, $\gamma _2 = - a_0 m$ and

$$\begin{aligned} \gamma _1&= \left( -\left\lfloor \frac{x}{m_0 n_0} + c_x(m - m_0)\right\rfloor q - r_0 + a_0 n_0\right) m. \end{aligned}$$

#### Proof

As in the proof of Lemma 4.3, we have

$$\begin{aligned} \left\{ \frac{x}{m_0 n_0} + c_x(m - m_0) + c_y(n - n_0)\right\}&= \left\{ \frac{r_0}{q} + \beta + \frac{a_0 (n-n_0)}{q} + \delta (n-n_0)\right\} \\&= \left\{ \beta + \delta (n-n_0)\right\} , \end{aligned}$$

where the last equality follows from the fact that $a_0(n-n_0) + r_0 \equiv 0 \pmod {q}.$ We know that $\beta + \delta (n-n_0)<1/q$, whereas $0<\textrm{ET}_{\textrm{quad}}(m,n)\le 1/2 b \le 1/q$. Since $q>1$, we see that, if $\beta + \delta (n-n_0) \ge 0$, the inequality

4.17 $$\begin{aligned} \left\lfloor \frac{x}{m n}\right\rfloor > \left\lfloor \frac{x}{m_0 n_0} + c_x(m - m_0) + c_y(n - n_0)\right\rfloor \end{aligned}$$

cannot hold. If $\beta + \delta (n-n_0) < 0$, then (4.17) holds iff

4.18 $$\begin{aligned} \frac{x}{m n} - \left( \frac{x}{m_0 n_0} + c_x(m - m_0) + c_y(n - n_0)\right) \ge - \beta - \delta (n-n_0), \end{aligned}$$

Much as in the proof of Lemma 4.3, this inequality holds iff $n\in I$, where $I= [\textbf{x}_-,\textbf{x}_+]$ if the equation $c_2 \textbf{x}^2 + c_1 \textbf{x} + c_0 = 0$ has real roots $\textbf{x}_-\le \textbf{x}_+$, where $c_0=x/m$, $c_2 = -a_0/q$ and

$$\begin{aligned} c_1 = -\left\lfloor \frac{x}{m_0 n_0} + c_x(m - m_0)\right\rfloor - \frac{r_0}{q} + \frac{a_0}{q} n_0 ,\end{aligned}$$

and $I=\emptyset$ if the equation has complex roots. $\square$

#### Lemma 4.6

Let $$(m,n)\in I_x\times I_y$$. If $$q=1$$,

$$\begin{aligned} L_2(m,n) - L_1(m,n) = {\left\{ \begin{array}{ll} 0 &{} \text {if }n \not \in (I_0\cap J) \cup (I_1\cap (\mathbb {R}\setminus J)),\\ 1 &{} \text {if }n \in (I_0\cap J) \cup (I_1\cap (\mathbb {R}\setminus J)), \end{array}\right. } \end{aligned}$$

where $J = n_0 -\beta /\delta -\frac{1}{\delta } (0,\infty )$ if $\delta \ne 0$, $J= \emptyset$ if $\delta =0$.

If $a\ne 0$, then $I_j = [\textbf{x}_{-,j},\textbf{x}_{+,j}]$ if the equation

$$\begin{aligned} \gamma _2 \textbf{x}^2 + \gamma _{1,j} \textbf{x} + \gamma _0 = 0 \end{aligned}$$

has real roots $\textbf{x}_{-,j}\le \textbf{x}_{+,j}$, and $I=\emptyset$ otherwise. Here $\gamma _0 = x q$, $\gamma _2 = - a_0 m$ and

$$\begin{aligned} \gamma _{1,j}&= \left( -\left\lfloor \frac{x}{m_0 n_0} + c_x(m - m_0)\right\rfloor q - (r_0+j) + a_0 n_0\right) m. \end{aligned}$$

If $$a=0$$, then

$$\begin{aligned} I_j = \left( -\infty , \frac{x}{m} \left( \left\lfloor \frac{x}{m_0 n_0} + c_x(m - m_0)\right\rfloor + r_0 + j \right) ^{-1} \right] . \end{aligned}$$

#### Proof

Just as in the proof of Lemma 4.5,

$$\begin{aligned} \left\{ \frac{x}{m_0 n_0} + c_x(m - m_0) + c_y(n - n_0)\right\} = \left\{ \beta + \delta (n-n_0)\right\} . \end{aligned}$$

If $\beta + \delta (n-n_0)<0$, then $L_2(m,n)-L_1(m,n)>0$ holds iff (4.18) holds. The term $\delta (n-n_0)$ cancels out, and so, by (4.6), we obtain that (4.18) holds iff

$$\begin{aligned} \frac{x}{m n} \ge \left\lfloor \frac{x}{m_0 n_0} + c_x(m - m_0)\right\rfloor + a_0 (n-n_0) + r_0, \end{aligned}$$

just as in Lemma 4.5. If $\beta + \delta (n-n_0)\ge 0$, $L_2(m,n)-L_1(m,n)>0$ holds iff (4.15) holds. Again, the term involving $\delta (n-n_0)$ cancels out fully, and so (4.18) holds iff

$$\begin{aligned} \frac{x}{m n} \ge \left\lfloor \frac{x}{m_0 n_0} + c_x(m - m_0)\right\rfloor + a_0 (n-n_0) + r_0 + 1. \end{aligned}$$

$$\square $$

In summary: for a neighborhood $I_x\times I_y$ small enough that $|\textrm{ET}_{\textrm{quad}}(m,n)|\le 1/2 b$, we need to prepare tables (in *O*(*b*) operations and space $O(b \log b)$) and compute a Diophantine approximation (in $O(\log b)$ operations). Then, for each value of *m*, we need to (i) compute $r_0=r_0(m)$, (ii) look up $\sigma _{r_0}$ in a table, (iii) solve a quadratic equation to account for the case $a_0 (n-n_0)+r_0\equiv -1 {{\,\mathrm{\,mod}\,}}q$, and (iv) solve a quadratic equation and also a linear equation to account for the case $a_0 (n-n_0) +r_0 \equiv 0 {{\,\mathrm{\,mod}\,}}q$. If $q=1$, then (iii) and (iv) are replaced by the simple task of computing the expressions in Lemma 4.6. In any event, there are a bounded number of tasks per *m*, each taking a bounded amount of (word) operations. Thus, the computation over the neighborhood $I_x \times I_y$ takes in total $O(a+b)$ word operations and space $O(b \log b)$, given the values of $\mu (m)$ and $\mu (n)$.

**Bitwise time bottleneck.** It should be evident that tasks (i), (iii) and (iv) above each take time on the order of $\log x \log \log x$; they involve multiplications, divisions and square roots of integers *N* with $\log N \asymp \log x$. Hence, the computation over $I_x\times I_y$ takes $\asymp (a+b) \log x \log \log x$ bit operations.

## Parameter choice. Final estimates

What remains now is to choose our neighborhoods $$U = I_x \times I_y$$ optimally (within a constant factor), and to specify our choice of *v*. Recall that $$I_x = [m_0-a,m_0+a)$$, $$I_y = [n_0-b,n_0+b)$$.

### Bounding the quadratic error term. Choosing *a* and *b*

We can use the formula for the error term bound in a Taylor expansion to obtain an upper bound on the error term. Since $f:(x,y)\mapsto X/x y$ is twice continuously differentiable for $x,y>0$, we know that, for (*x*, *y*) in any convex neighborhood *U* of any $(x_0,y_0)$ with $x_0,y_0>0$,

$$\begin{aligned} \frac{X}{x y} = \frac{X}{x_0 y_0} + \frac{\partial f(x_0,y_0)}{\partial x} (x - x_0) + \frac{\partial f(x_0,y_0)}{\partial y} (y-y_0) + \textrm{ET}_{\textrm{quad}}(x,y), \end{aligned}$$

where the *Lagrange remainder term* $\textrm{ET}_{\textrm{quad}}(x,y)$ is given by

$$\begin{aligned} \begin{aligned} \textrm{ET}_{\textrm{quad}}(x,y)&= \frac{1}{2} \frac{\partial ^2 f(\xi ,\upsilon )}{\partial ^2 x} (x-x_0)^2 + \frac{1}{2} \frac{\partial ^2 f(\xi ,\upsilon )}{\partial ^2 y} (y-y_0)^2 \\ {}&+ \frac{\partial ^2 f(\xi ,\upsilon )}{\partial x \partial y} (x-x_0) (y-y_0), \end{aligned} \end{aligned}$$

for some $(\xi ,\upsilon )=(\xi (x,y),\upsilon (x,y))\in U$ depending on (*x*, *y*). Working with our neighborhood $U=I_x\times I_y$ of $(x_0,y_0)=(m_0,n_0)$, we obtain that, for $m\in I_x$ and $n\in I_y$, $|\textrm{ET}_{\textrm{quad}}(m,n)|$ is at most

5.1 $$\begin{aligned}&\le \frac{X}{m'^3 n'}(m - m_0)^2 + \frac{X}{m'^2 n'^2} (m-m_0) (n-n_0) + \frac{X}{m'n'^3}(n - n_0)^2,\end{aligned}$$

where $m' = \min _{(m, n) \in U} m$ and $n' = \min _{(m, n) \in U} n.$ Hence, by Cauchy-Schwarz,

$$\begin{aligned} |\textrm{ET}_{\textrm{quad}}(m,n)|\le \frac{3}{2} \left( \frac{X}{m'^3 n'}(m - m_0)^2 + \frac{X}{m'n'^3}(n - n_0)^2\right) . \end{aligned}$$

(From now on, we will write *x*, as we are used to, instead of *X*, since there is no longer any risk of confusion with the variable *x*.)

Recall that we need to choose $I_x$ and $I_y$ so that $\left| \text {ET}_{\text {quad}}\right| \le 1/2 b$. Since $(m - m_0)^2 \le a^2$ and $(n - n_0)^2 \le b^2$, it is enough to require that

$$\frac{x}{m'^3 n'} a^2 \le \frac{1}{6 b},\;\;\; \ \frac{x}{m' n'^3} b^2 \le \frac{1}{6 b}.$$

In turn, these conditions hold for

$$a = \root 3 \of {\frac{(m')^4}{6 x}},\;\;\; b = \root 3 \of {\frac{m' (n')^3}{6 x}}.$$

More generally, if we are given that $m'\ge A$, $n'\ge B$ for some *A*, *B*, we see that we can set

5.2 $$\begin{aligned} a = \root 3 \of {\frac{A^4}{6 x}},\;\;\; b = \root 3 \of {\frac{A B^3}{6 x}}.\end{aligned}$$

At the end of Sect. 4, we showed that it takes $O(a+b)$ operations and space $O(b \log b)$ for our algorithm to run over each neighborhood $I_x \times I_y$. Recall that we are dividing $[1, v] \times [1,v]$ into dyadic boxes (or, at any rate, boxes of the form $\textbf{B}(A,B,\eta ) = [A,(1+\eta ) A) \times [B,(1+\eta ) B)$, where $0<\eta \le 1$ is a constant) and that these boxes are divided into neighborhoods $I_x \times I_y$. We have $\ll \frac{A B}{ab}$ neighborhoods $I_x \times I_y$ in the box $\textbf{B}(A,B,\eta )$. Thus, assuming that $A\ge B$, it takes

$$O\left( \frac{A B}{a b}(a + b)\right) = O\left( \frac{A B}{b}\right) = O\left( A^{2/3} x^{1/3}\right) $$

operations to run over this box, using the values of *a* and *b* in (5.2).

Now, we will need to sum over all boxes $\textbf{B}(A,B,\eta )$. Each *A* is of the form $\lceil (1+\eta )^i\rceil$ and each *B* is of the form $\lceil (1+\eta )^j\rceil$ for $1 \le (1+\eta )^i, (1+\eta )^j \le v.$ By symmetry, we may take $j\le i$, that is, $A\ge B$. Summing over all boxes takes

$$\begin{aligned} \begin{aligned} \ll \sum _{i:(1+\eta )^i \le v} \sum _{j\le i} ((1+\eta )^i)^{2/3} x^{1/3}&\ll \sum _{i:(1+\eta )^i \le v} i ((1+\eta )^i)^{2/3} x^{1/3} \\ \ll (\log v) v^{2/3} x^{1/3}&\le v^{2/3} x^{1/3} \log x\end{aligned} \end{aligned}$$

operations.

We tacitly assumed that $a\ge 1$, $b\ge 1$, and so we need to handle the case of $a<1$ or $b<1$ separately, by brute force. It actually makes sense to treat the broader case of $a<C$ or $b<C$ by brute force, where *C* is a constant of our choice. The cost of brute-force summation for (*m*, *n*) with $n\le m\ll (C^3 x)^{1/4}$ (as is the case when $a<C$) is

$$\begin{aligned} \ll ((6 C^3 x)^{1/4})^2 \ll x^{1/2}, \end{aligned}$$

whereas the cost of brute-force summation for (*m*, *n*) with $m\le v$, $n\ll (6 x/m)^{1/3}$ (as is the case when $b<C$) is

$$\begin{aligned} \ll \sum _{m\le v} \frac{x^{1/3}}{m^{1/3}} \ll x^{1/3} v^{2/3}. \end{aligned}$$

Lastly, we need to take into account the fact that we had to pre-compute a list of values of $\mu$ using a segmented sieve (Algorithm 20), which takes $O(v^{3/2} \log \log x)$ operations and space $O(\sqrt{v} \log \log v)$. Putting everything together, we see that the large free variable case (Sect. 4) takes

$$\begin{aligned}{} & {} O(v^{2/3} x^{1/3} \log x + v^{3/2} \log \log x)\;\;\;\; \text {operations and}\\{} & {} \quad \text {space}\;\;\;\; O(\sqrt{v} \log \log x + (v^4/x)^{1/3} \log x), \end{aligned}$$

where the space bound comes from substituting $b = \root 3 \of {\frac{m' (n')^3}{6 x}}$ into the space estimate that we had for each neighborhood and adding it to the space bound from the segmented sieve.

### Choice of *v*. Total time and space estimates

Recall that the case of a large non-free variable (Algorithm 2) takes $O((\frac{x}{v} + u) \log \log x)$ operations and space $O(\sqrt{\max (x/v, u)} \log x)$. At the end of Sect. 3, we took $u=\sqrt{x}$, resulting in $O(\frac{x}{v} \log \log x)$ operations and space $O(\sqrt{x/v} \log x)$.

On the other hand, as we just showed, the case of a large free variable (Algorithm 5) takes $O(v^{2/3} x^{1/3} \log x + v^{3/2} \log \log x)$ operations and space $O(\sqrt{v} \log \log x + (v^4/x)^{1/3} \log x)$.

Thus, in order to minimize our number of operations, we set the two time bounds equal to one another and solve for *v*, yielding

$$\begin{aligned} v = x^{2/5} (\log \log x)^{3/5}/(\log x)^{3/5}. \end{aligned}$$

Using this value of *v* (or any value of *v* within a constant factor *c* of it) allows us to obtain

$$\begin{aligned}{} & {} O\left( x^{\frac{3}{5}} (\log x)^{\frac{3}{5}} (\log \log x)^{\frac{2}{5}} \right) \ \ \textrm{operations} \ \ \textrm{and}\\{} & {} \textrm{space} \ \ O\left( x^{\frac{3}{10}} (\log x)^{\frac{13}{10}} (\log \log x)^{-\frac{3}{10}} \right) , \end{aligned}$$

as desired. Note that our algorithm for the case of a large non-free variable uses more memory, by far, than that for the case of a large free variable.

The resulting number of bit operations is

$$\begin{aligned} O\left( x^{\frac{3}{5}} (\log x)^{\frac{3}{5}} (\log \log x)^{\frac{2}{5}} \right) \cdot O(\log x \log \log x) = O\left( x^{\frac{3}{5}} (\log x)^{\frac{8}{5}} (\log \log x)^{\frac{7}{5}} \right) . \end{aligned}$$

We already explained (at the end of Sects. 3 and 4) that one cannot really hope for a factor better than $O(\log x \log \log x)$ here, given our current algorithm.

The constant *c* can be fine-tuned by the user or programmer. It is actually best to set it so that the time taken by the case of a large free variable and by the case of a large non-free variable are within a constant factor of each other without being approximately equal.

If we were to use [5] to factor integers in SArr (Algorithm 4) then LargeNonFree (Algorithm 2) would take $O((x/v) \log x)$ operations and space $O((x/v)^{1/3} (\log (x/v))^{5/3})$. It would then be best to set $v = c\cdot x^{2/5}$ for some *c*, leading to $O(x^{3/5} \log x)$ operations in total and total space $O\left( x^{1/5} (\log x)^{5/3}\right)$.

## Implementation details

We wrote our program in C++ (though mainly simply in C). We used gmp (the GNU MP multiple precision library) for a few operations, but relied mainly on 64-bit and 128-bit arithmetic. Some key procedures were parallelized by means of OpenMP pragmas.

*Basics on better sieving.* Let us first go over two well-known optimization techniques. The first one is useful for sieving in general; the second one is specific to the use of sieves to compute $\mu (n)$.

1. When we sieve (function SegPrimes, SegMu or SegFactor), it is useful to first compute how our sieve affects a segment of length $M=2^3\cdot 3^2\cdot 5\cdot 7\cdot 11$, say. (For instance, if we are sieving for primes, we compute which elements of $\mathbb {Z}/M\mathbb {Z}$ lie in $(\mathbb {Z}/M \mathbb {Z})^*$.) We can then copy that segment onto our longer segment repeatedly, and then start sieving by primes and prime powers not dividing *M*.
2. As is explained in [9] and [6], and for that matter in [4, § 4.5.1]: in function SegMu, for $n\le x_0 = n_0 + \Delta$, we do not actually need to store $\Pi _j = \sum _{p\le \sqrt{x_0}: p|n} p$; it is enough to store $S_j \sum _{p\le \sqrt{x_0}} \lceil \log _4 p\rceil$. The reason is that (as can be easily checked) $\Pi _j < \prod _{p|n} p$ if and only if $S_j < \lceil \log _4 n\rceil$. In this way, we use space $O(\Delta \log \log x_0)$ instead of space $O(\Delta \log x_0)$. We also replace many multiplications by additions; in exchange, we need to compute $\lceil \log _4 p\rceil$ and $\lceil \log _4 n\rceil$, but that takes very little time, as it only involves counting the space occupied by *p* or *n* in base 2, and that is a task that a processor can usually accomplish extremely quickly.

Technique (2) here is not essential in our context, as SegMu is not a bottleneck, whether for time or for space. It is more important to optimize factorization – as we are about to explain.

*Factorizing via a sieve in little space.* We wish to store the list of prime factors of a positive number *n* in at most twice as much space as it takes to store *n*. We can do so simply and rapidly as follows. We initialize $a_n$ and $b_n$ to 0. When we find a new prime factor *p*, we reset $a_n$ to $2^k a_n + 2^{k-1}$, where $k = \lfloor \log _2 p\rfloor$, and $b_n$ to $2^k b_n + p - 2^k$. In the end, we obtain, for example,

$$a_{2\cdot 3\cdot 5\cdot 7} = 111010_2,\;\; b_{2\cdot 3\cdot 5\cdot 7} = 010111_2.$$

We can easily read the list of prime factors 2, 3, 5, 7 of $n=2\cdot 3\cdot 5\cdot 7$ from $a_n$ and $b_n$, whether in ascending or in descending order: we can see $a_n$ as marking where each prime in $b_n$ begins, as well as providing the leading 1: $2=\textbf{1}0_2$, $3=\textbf{1}1_2$, $5=\textbf{1}01_2$, $7=\textbf{1}11_2$.

The resulting savings in space lead to a significant speed-up in practice, due no doubt in part to better cache usage. The bitwise operations required to decode the factorization of *n* are very fast, particularly if one is willing to go beyond the *C* standard; we used instructions available in gcc (__builtin_clzl, __builtin_ctzl).

*Implementing the algorithm in integer arithmetic.* Manipulating rationals is time consuming in practice, even if we use a specialized library. (Part of the reason is the frequent need to reduce fractions *a*/*b* by taking the $\gcd$ of *a* and *b*.) It is thus best to implement the algorithm – in particular, procedure SumByLin and its subroutines – using only integer arithmetic. Doing so also makes it easier to verify that the integers used all fit in a certain range ($|n|<2^{127}$, say), and of course also helps them fit in that range, in that we can simplify fractions before we code: (*a*/*bc*)/(*d*/*bf*) (say) becomes *af*/*bd*, represented by the pair of integers (*af*, *bd*).

*Square-roots and divisions.* On typical current 64-bit architectures, a division takes as much time as several multiplications, and a square-root takes about as much time as a division. (These are obviously crude, general estimates.) Here, by “taking a square-root” of *x* we mean computing the representable number closest to $\sqrt{x}$, or the largest representable number no larger than $\sqrt{x}$, where “representable” means “representable in extended precision”, that is, as a number $2^e n$ with $|n|<2^{128}$ and $e \in [-(2^{14}-1),2^{14}-1] - 63$.

Incidentally, one should be extremely wary of using hardware implementations of any floating-point operations other than the four basic operations and the square-root; for instance, an implementation of $\exp$ can give a result that is *not* the representable number closest to $\exp (x)$ for given *x*. Fortunately, we do not need to use any floating-point operations other than the square-root. The IEEE 754 standard requires that taking a square-root be implemented correctly, that is, that the operation return the representable number closest to $\sqrt{x}$, or the largest representable number $\le \sqrt{x}$, or the smallest such number $\ge \sqrt{x}$, depending on how we set the rounding mode.

We actually need to compute $\lfloor \sqrt{n}\rfloor$ for *n* a 128-bit integer. (We can assume that $n<2^{125}$, say.) We do so by combining a single iteration of the procedure in [21] (essentially Newton’s method) with a hardware implementation of a floating-point extended-precision square-root in the sense we have just described.

It is of course in our interest to keep the number of divisions (and square-roots) we perform as low as possible; keeping the number of multiplications small is of course also useful. Some easy modifications help: for instance, we can conflate functions Special1 and Special0B into a single procedure; the value of $\gamma _1$ in the two functions differs by exactly *m*.

*Parallelization.* We parallelized the algorithm at two crucial places: one is function SArr (Algorithm 4), as we already discussed at the end of Sect. 3; the other one is function DDSum (Algorithm 7), which involves a double loop. The task inside the double loop (that is, DoubleSum or BruteDoubleSum) is given to a processing element to compute on its own. How exactly the double loop is traversed and parcelled out is a matter that involves not just the usual trade-off between time and space but also a possible trade-off between either and efficiency of parallelization.

More specifically: it may be the case that the number of processing elements is greater than the number of iterations of either loop ($\lceil (A'-A)/\Delta \rceil$ and $\lceil (B'-B)/\Delta \rceil$, respectively), but smaller than the number of iterations of the double loop. In that case, parallelizing only the inside loop or the outside loop leads to an under-utilization of processing elements. One alternative is a naïve parallelization of the double loop, with each processing element recomputing the arrays $\mu$, $\mu '$ that it needs. That actually turns out to be a workable solution: while recomputing arrays in this way is wasteful, the overall time complexity does not change, and the total space used is $O(\nu \Delta \log \log \max (A',B'))$, where $\nu$ is the number of threads; this is slightly less space than $\nu$ instances of SumbyLin use anyhow.

The alternative of computing and storing the whole arrays $\mu$, $\mu '$ before entering the double loop would allow us not to recompute them, but it would lead to using (shared) memory on the order of $\max (A',B') \log \log \max (A',B')$, which may be too large. Yet another alternative is to split the double loop into squares of side about $\sqrt{\nu } \Delta$; then each array segment $\mu$, $\mu '$ is recomputed only about $(A'-A)/(\sqrt{\nu } \Delta )$ or $(B'-B)/(\sqrt{\nu } \Delta )$ times, respectively, and we use $O(\sqrt{\nu } \Delta )$ shared memory. Our implementation of this last alternative, however, led to a significantly worse running time, at least for $x=10^{19}$; in the end, we went with the “workable solution” above. In the end, what is best may depend on the parameter range and number of threads one is working with.

## Numerical results

We computed *M*(*x*) for $x=10^n$, $n\le 23$, and $x=2^n$, $n\le 75$, beating the records in [9] and [6]. Our results are the same as theirs, except that we obtain a sign opposite to that in [9, Table 1] for $x=10^{21}$; presumably [9] contains a transcription mistake. 
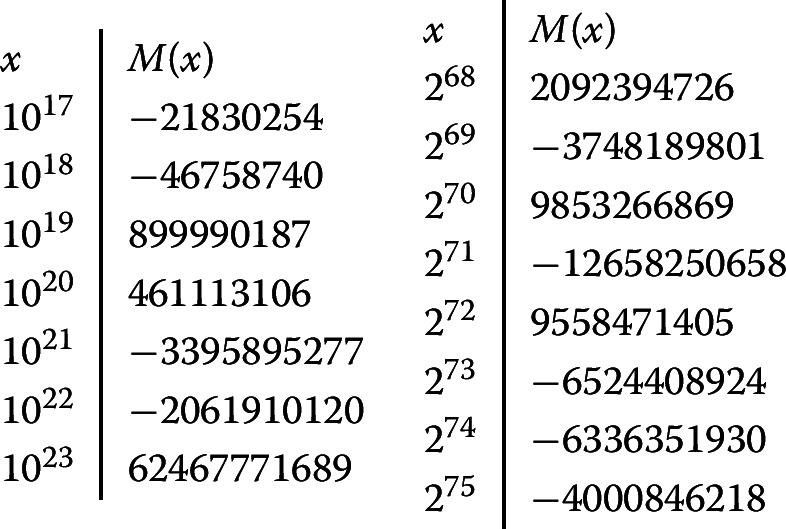


Computing *M*(*x*) for $x=10^{23}$ took about 18 days and 14.6 hours on a 80-core machine (Intel Xeon 6148, 2.40 GHz) shared with other users. Computing *M*(*x*) for $x=2^{75}=3.777\dotsc \cdot 10^{22}$ took about 9 days and 16 hours on the same machine. (These are wall times, not CPU times.) As we shall see shortly, one parameter *c* was more strictly constrained for $x=10^{23}$, since we needed to avoid overflow; we were able to optimize *c* more freely for $2^{75}$.

**Fig. 1:**
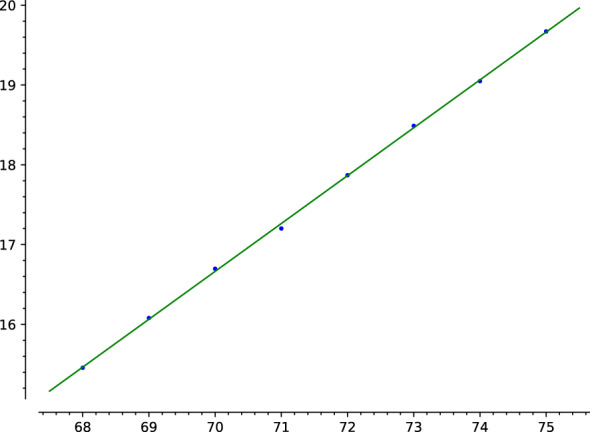
Logarithm base 2 of running time for input $x=2^n$

For a fixed choice of parameters, running time scaled approximately as $x^{3/5}$. See Fig. 1 for a plot^2^ of the logarithm base 2 of the running time (in seconds; wall time) for $x= 2^n$, $n=68,69,\dotsc ,75$ with $v= x^{2/5}/3$. We have drawn a line of slope 3/5, with constant coefficient chosen by least squares to fit the points with $68\le n\le 75$.

We also ran our code for $x=2^n$, $68\le n\le 75$, on a 128-core machine based on two AMD EPYC 7702 (2GHz) processors. The results were of course the same as on the first computer, but running time scaled more poorly, particularly when passing from $2^{73}$ to $2^{74}$. (For whatever reason, the program gave up on $n=2^{75}$ on the second computer.) The percentage of total time taken by the case of a large non-free variable was also much larger than on the first computer, and went up from $2^{73}$ to $2^{74}$. The reason for the difference in running times in the two computers presumably lies in the differences between their respective memory architectures. The dominance (in the second computer) of the case of a large non-free variable, whose usage of sieves is the most memory-intensive part of the program, supports this diagnosis. It would then be advisable, for the sake of reducing running times in practice, to improve on the memory usage of that part of the program, either replacing SegFactor by the improved sieve in [5] – sharply reducing memory usage at the cost of increasing the asymptotic running time slightly, as we have discussed – or using a cache-efficient implementation of the traditional segmented sieve as in [15, Algorithm 1.2]. These two strategies could be combined.

*Checking for overflow.* Since our implementation uses 128-bit signed integers, it is crucial that all integers used be of absolute value $<2^{127}$. What is critical here is the quantity

$$\begin{aligned} \frac{\beta }{\delta } = \frac{(\overline{x (m_\circ - (m-m_\circ ))} /m_\circ ^2 n_\circ - r_0/q}{ - x/m_\circ n_\circ ^2 - a/q} = \frac{(\overline{x (2 m_\circ - m)} q - r_0 m_\circ ^2 n_\circ ) n_\circ }{(-x q - a m_\circ n_\circ ^2) m_\circ } \end{aligned}$$

in SumByLim, where we write here $\overline{y}$ for the integer in $\{0,1,\dotsc , m_\circ ^2 n_{\circ }-1\}$ congruent to *y* modulo $m_\circ ^2 n_\circ$. The numerator could be as large as $q m_\circ ^2 n_\circ ^2$ (The denominator is much smaller, since $|-x/m_\circ n_\circ ^2 - a/q|\le 1/2 b q$.) Since $q\le 2 b$, $b\le (A^4/6 x)^{1/3} \le (v^4/6 x)^{1/3}$, $m_\circ ,n_\circ \le v$ and $v = c x^{2/5} \frac{(\log \log x)^{3/5}}{(\log x)^{3/5}}$, we see that

7.1 $$\begin{aligned} q m_\circ ^2 n_\circ ^2\le \frac{2 v^{16/3}}{(6 x)^{1/3}} = \frac{ 2 c^{16/3}}{6^{1/3}} \cdot x^{9/5} \frac{(\log \log x)^{\frac{16}{5}}}{(\log x)^{\frac{16}{5}}} . \end{aligned}$$

For $$c=3/2$$ and $$x=2^{75} = 3.777\dotsc \cdot 10^{22}$$,

$$\begin{aligned} \log _2 \left( \frac{ 2 c^{16/3}}{6^{1/3}} x^{9/5} \frac{(\log \log x)^{\frac{16}{5}}}{(\log x)^{\frac{16}{5}}}\right) = 126.361\dotsc < 127; \end{aligned}$$

for $$c = 9/8$$ and $$x=10^{23}$$,

$$\begin{aligned} \log _2 \left( \frac{ 2 c^{16/3}}{6^{1/3}} x^{9/5} \frac{(\log \log x)^{\frac{16}{5}}}{(\log x)^{\frac{16}{5}}}\right) = 126.611\dotsc < 127. \end{aligned}$$

Thus, our implementation should give a correct result for $x = 10^{23}$, for the choice $c=9/8$. One can obviously go farther by using wider (or arbitrary-precision) integer types.

There is another integer that might seem to be possibly larger, namely the discriminant $\Delta = b^2-4 a c$ in the quadratic equations solved in QuadIneqZ, which is called by functions Special1 and Special0B. However, that discriminant is smaller than it looks at first.

The coefficient $\gamma _1$ in Special0B is

$$\begin{aligned} \begin{aligned}&(-\lfloor R_0\rfloor q - r_0 + a_0 n_\circ ) m = (-\lfloor R_0\rfloor q - (\{R_0\} - \beta ) q + a_0 n_\circ ) m \\ {}&\quad = \left( - \left( \frac{x}{m_\circ n_\circ } - \frac{x}{m_\circ ^2 n_\circ } (m-m_\circ ) \right) q + \beta q + a_0 n_\circ \right) m\\&\quad = \left( - \left( \frac{x}{m_\circ n_\circ } - \frac{x}{m_\circ ^2 n_\circ } (m-m_\circ ) \right) + \beta + \left( -\frac{x}{m_\circ n_\circ ^2} - \delta \right) n_\circ \right) m q\\&\quad = \left( -\frac{2 x}{m_\circ n_\circ } + \frac{x (m-m_\circ )}{m_\circ ^2 n_\circ } + O^*\left( \frac{1}{2 q}\right) + O^*\left( \frac{1}{2 b q}\right) n_\circ \right) m q . \end{aligned} \end{aligned}$$

Here the second term is negligible compared to the first one, and the third term is negligible compared to the fourth one. We know that

$$\begin{aligned} \begin{aligned} \frac{x}{m_\circ n_\circ } m q&\le \frac{x}{m_\circ n_\circ } (m_\circ +a) \cdot 2 b \le \frac{2 b x}{n_\circ } + \frac{2 a b x}{m_\circ n_\circ }\\&\le 2 x \root 3 \of {\frac{A}{6 x}} + 2 x \root 3 \of {\frac{A^2}{(6 x)^2}} \le 2 x \root 3 \of {\frac{v}{6 x}} + 2 x \root 3 \of {\frac{v^2}{(6 x)^2}}\\&\le 2 \root 3 \of {\frac{c}{6}} \cdot x^{\frac{4}{5}} \left( \frac{\log \log x}{\log x}\right) ^{1/5} + 2 \left( \frac{c}{6}\right) ^{\frac{2}{3}} x^{\frac{3}{5}} \left( \frac{\log \log x}{\log x}\right) ^{2/5}. \end{aligned} \end{aligned}$$

We also see that

$$\begin{aligned} \frac{n_\circ m}{2 b} \le \frac{n_\circ m_\circ }{b} \le \root 3 \of {6 x \cdot A^2} \le \root 3 \of {6 v^2 x} \le \root 3 \of {6 c^2}\cdot x^{\frac{3}{5}} \left( \frac{\log \log x}{\log x}\right) ^{2/5} . \end{aligned}$$

The dominant term is thus $2 (c/6)^{1/3} x^{4/5} ((\log \log x)/\log x)^{1/5}$. The coefficient $\gamma _1$ in Special1 is equal to the one we just considered, minus *m*, and thus has the same dominant term.

As for the term $- 4 a c$ (or $- 4 \gamma _0 \gamma _2$, so as not to conflict with the other meanings of *a* and *c* here), it equals 4 times

$$\begin{aligned} a m x q = \frac{a}{q} m x q^2 = \left( -\frac{x}{m_\circ n_\circ ^2} - \delta \right) m x q^2 = - \frac{x^2 q^2 m}{m_\circ n_\circ ^2} + O^*(m x). \end{aligned}$$

Since

$$\begin{aligned} \frac{x^2 q^2}{n_\circ ^2} \le \frac{4 x^2 b^2}{B^2} = 4 x^2 \root 3 \of {A^2}{(6 x)^2} \le \frac{4}{6^{2/3}} x^{4/3} v^{2/3} \le \frac{4 c^{2/3}}{6^{2/3}} x^{8/5} \left( \frac{\log \log x}{\log x}\right) ^{2/5} \end{aligned}$$

and $m x\le v x \le c x^{7/5} (\log \log x)^{3/5}/(\log x)^{3/5}$, we see that the main term here is at most

$$\begin{aligned} \frac{16 c^{2/3}}{6^{2/3}} x^{8/5} \left( \frac{\log \log x}{\log x}\right) ^{2/5}. \end{aligned}$$

Since the two expressions we have just considered have opposite sign, we conclude that the main term in the discriminant $\gamma _1^2 - 4 \gamma _0 \gamma _2$ is thus at most $(16 c^{2/3}/6^{2/3}) x^{8/5} (\log \log x)^{2/5}/(\log x)^{2/5}$, that is, considerably smaller than the term in (7.1), at least for *x* larger than a constant. For $c=3/2$ and $x=2^{75}$,

$$\begin{aligned} \log _2 \frac{16 c^{2/3}}{6^{2/3}} x^{8/5} \left( \frac{\log \log x}{\log x}\right) ^{2/5} = 121.179\dotsc . \end{aligned}$$

For $$c=9/8$$ and $$x=10^{23}$$,

$$\begin{aligned} \log _2 \frac{16 c^{2/3}}{6^{2/3}} x^{8/5} \left( \frac{\log \log x}{\log x}\right) ^{2/5} = 123.141\dotsc , \end{aligned}$$

and thus we are out of danger of overflow for those parameters as well.

## Figures and Tables

**Fig. 2:**
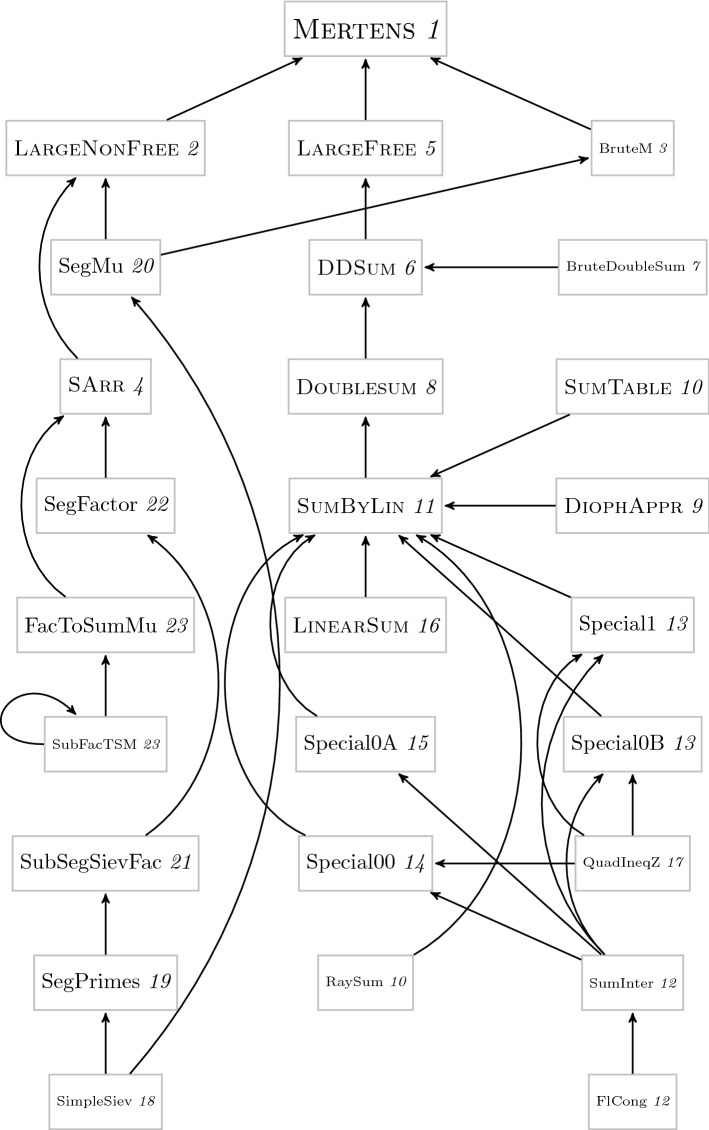
Dependency diagram for Mertens. Function names are followed by algorithm number

## Data Availability

All data generated or analysed during this study are included in this published article.

## Appendix A: A sketch of an alternative algorithm

As we mentioned in the introduction, we originally developed an algorithm taking $O(x^{3/5} (\log x)^{8/5})$ word operations and space $O(x^{3/10} \log x)$, or, if the sieve in [5] is used to factorize integers in function SArr (Algorithm 4), $O(x^{3/5} (\log x)^{8/5})$ word operations and space $O(x^{1/5} (\log x)^{1/5+5/3})$. The algorithm actually had an idea in common with [5]; as explained there, it is an idea inspired by Voronoï and Vinogradov’s approach to the divisor problem.

Part of the improvement over that older algorithm resides in a better (yet simple) procedure for computing sums of the form $$\sum _{d|n: d\le a} \mu (d)$$ (see Algorithm 23); we analyzed it in Sect. 3. Other than that, the difference lies mainly in the computation of the sum of $$\mu (m) \mu (n) \lfloor x/m n\rfloor $$ for (*m*, *n*) in a neighborhood $$U = I_x\times I_y$$ (see Sect. 4.2 and Algorithm 11). Let us use the notation in §4.2. In particular, write $$I_x = [m_0-a,m_0+a)$$, $$I_y = [n_0-b,n_0+b)$$. We have sums $$S_0$$, $$S_1$$, $$S_2$$, where $$S_0$$ is easy to compute and $$S_2$$ is the sum that we actually want to determine.

In the algorithm given in the current version of the paper, we compute the difference $S_1-S_0$ in $O(a+b)$ operations and space $O(b \log b)$. Computing the difference $S_1-S_0$ in $O((a+b) \log b)$ operations and space $O(b \log b)$ (as we did in a previous version of the paper) is not actually hard; the main steps are: (i) sort the list of all pairs $(\{c_y (n-n_0)\},n)$ by their first element $\{c_y (n-n_0)\}$, (ii) use the sorted list to compute the sums $\sum _{n: \{c_y n\}\ge \{c_y n'\}} \mu (n)$ for different $n'$, and then (iii) search through the list as needed to determine the sum $\sum _{n:\{c_y n\}\ge \beta } \mu (n)$ for any given value of $\beta$.

The crux is how to compute $S_2-S_1$. In the current version, we analyze this difference with great care, after having determined the (at most) two arithmetic progressions in which the terms of $S_2-S_1$ that are non-zero must be contained. In the older version, we determined those arithmetic progressions in the same way as here (namely, by finding a Diophantine approximation *a*/*q* to $c_y$). Within those progressions, however, we did not establish precisely what the non-zero terms were, but simply showed that they had to be contained in an interval $I\subset I_y$. We also showed that, for *q* small, the interval *I* had to be small as well, at least on average. (The number of elements of an arithmetic progression modulo *q* within $I_y$ is *O*(*b*/*q*), and so the case of *q* large is not the main worry.) It is here that the argument in [20, Ch. III, exer. 3-6] came in handy: as we move from neighborhood to neighborhood, the quantity $c_y$ keeps changing at a certain moderate speed, monotonically; thus, $c_y {{\,\mathrm{\,mod}\,}}\mathbb {Z}$ cannot spend too much time in major arcs on the circle $\mathbb {R}/\mathbb {Z}$. Only when $c_y {{\,\mathrm{\,mod}\,}}\mathbb {Z}$ lies in the major arcs can *q* be small and the interval *I* be large. Thus, just as claimed, the case of *q* small and *I* large occurs for few neighborhoods.

We can thus simply determine *I*, and compute the terms that lie in the intersection of either of those two arithmetic progressions and their corresponding intervals *I*, and sum those terms. The number of operations will be about *O*(*ab*/*q*), unless *q* is small, in which case one can do better, viz., *O*(*a*|*I*|/*q*) or so. (Compare with the corresponding bound for the newer algorithm, namely, $O(a+b)$.) On average, we obtained savings of a factor of $O((\log b)/b)$, rather than *O*(1/*b*), as we do now.

Whether or not we use [5] to factor integers $n\le x/v$, we set $v = c x^{2/5}/(\log x)^{3/5}$, for *c* a constant of our choice.

## Appendix B: Pseudocode for algorithms

We will now give the pseudocode for the algorithms referenced in this paper. To aid the reader, we include a diagram demonstrating the relationship between the algorithms. As before, “operations” means “word operations”, i.e., “arithmetic operations ($+$,−,$\cdot$,/,$\surd$) on integers *n* up to $x^{O(1)}$” and “reading and writing such integers in arrays of size $x^{O(1)}$”.

## Footnotes

1 One may take a little more space (but no more than $O(\sqrt{x/v} \log (x/v))$) if one decides to parallelize this summation procedure.

2 The first time we ran the program for $x=2^{75}$, we obtained a substantially higher running time, on the order of fourteen and a half days (as was reported on the first public draft of this paper). The time taken for $x=2^{71}$ was also higher on a first run, by about 20%. We do not know the reason for this discrepancy, though demands by other users are probably the reason for $x=2^{71}$ and possibly also for $x=2^{75}$.
